# A randomized controlled efficacy trial of behavioral activation for concurrent stimulant use and sexual risk for HIV acquisition among MSM: project IMPACT study protocol

**DOI:** 10.1186/s12889-018-5856-0

**Published:** 2018-07-25

**Authors:** Matthew J. Mimiaga, David W. Pantalone, Katie B. Biello, Tiffany Rose Glynn, Christopher M. Santostefano, Jennifer Olson, Dana J. Pardee, Jaclyn M. W. Hughto, Josibel Garcia Valles, Adam W. Carrico, Kenneth H. Mayer, Steven A. Safren

**Affiliations:** 1 Center for Health Equity Research, Brown University, 121 South Main Street, Providence, RI 02903 USA; 2 Departments of Behavioral & Social Health Sciences and Epidemiology, Brown University, School of Public Health, Providence, RI USA; 3 Department of Psychiatry and Human Behavior, Brown University Alpert Medical School, Providence, RI USA; 4 The Fenway Institute, Fenway Health, Boston, MA USA; 5 Department of Psychology, University of Massachusetts, Boston, MA USA; 6 Department of Psychology, University of Miami, Coral Gables, FL USA; 7 Department of Public Health Sciences, University of Miami Medical School, Coral Gables, FL USA; 8 Department of Infectious Diseases, Harvard Medical School/Beth Israel Deaconess Medical Center, Boston, MA USA; 9 Department of Global Health and Population, Harvard T.H. Chan School of Public Health, Boston, MA USA

**Keywords:** HIV, Sexual risk, Men who have sex with men (MSM), Stimulant use, Behavioral activation

## Abstract

**Background:**

In the United States, problematic stimulant use is a prevalent and difficult to treat problem among men who have sex with men (MSM), as well as a major driver of HIV transmission through the large number of sexual partners and concomitant condomless anal sex (CAS). Evidence-based behavioral studies that address problematic stimulant use in MSM at risk for HIV infection are also lacking. In this paper, we describe the design of a behavioral intervention trial to reduce sexual risk behavior and stimulant use in HIV-uninfected MSM.

**Methods:**

This study, funded by the National Institute on Drug Abuse (NIDA), is a randomized controlled trial (RCT) testing an integrated HIV risk reduction and behavioral activation counseling intervention (IMPACT) for HIV-uninfected, stimulant using MSM in Boston, MA, and Miami, FL. Participants are randomized (2:2:1) to either (1) the IMPACT intervention; (2) a relaxation condition, an active therapy time- and intensity-matched control; or (3) a standard of care risk reduction counseling comparison. At enrollment, all participants receive an HIV test and pre- and post-test counseling. The primary outcome is the difference in the rate of change in the number of self-reported condomless anal sex acts without the protection of consistent Pre-Exposure Prophylaxis (PrEP) use, as well as reductions in stimulant use during the prior 4-months. Major assessments are conducted at baseline, 4-, 8-, and 12-month follow-up visits.

**Discussion:**

Effective and sustainable behavioral interventions are sorely needed to reduce HIV acquisition in stimulant using MSM at risk for HIV infection. In this study, we will evaluate the evidence of efficacy of the IMPACT intervention to reduce HIV acquisition in HIV-uninfected, stimulant-using MSM. If found effective, the intervention tested here holds promise for being readily integrated into real-world clinical settings.

**Trial registration:**

ClinicalTrials.gov number NCT03175159, registered June 5, 2017.

## Background

Men who have sex with men (MSM) continue to be, by far, the largest risk group for HIV infection in the United States. Although the annual number of HIV diagnoses among Americans declined 5% between 2010 and 2015, MSM are the largest exposure category [1]. In 2015, MSM represented 70% of new HIV infections diagnosed in the U.S. despite comprising only 2% of the population [2]. Stimulant use (i.e., crystal methamphetamine [meth], cocaine, crack) is also endemic to urban American MSM [3–6]. The prevalence of stimulant use among MSM has been shown to be 20 times that of the general population, with an estimated 10–25% of MSM reporting use of stimulants in the context of sexual behavior in the past 6 months [7–12]. Epidemiological studies with U.S. MSM demonstrate that problematic stimulant use has remained steady [13–15]. Stimulants remain relatively affordable, easy to manufacture, and readily available [16] maintaining a broad and negative health impact nationwide [17–21].

Problematic stimulant use and HIV infection are intertwined epidemics affecting MSM. Findings from a number of studies suggest that stimulant use is a major driver of HIV transmission in MSM, as stimulant-using MSM report higher numbers of sexual partners and are more likely to endorse condomless anal sex (CAS) than non-stimulant using peers [22–28]. Given research showing that the primary medical correlate of stimulant use disorder is HIV infection [29], HIV-uninfected MSM who use stimulants at threshold diagnostic levels of the fifth edition of the Diagnostic and Statistical Manual (DSM-5) are at exceptionally high risk for HIV acquisition via drug-associated sexual risk behaviors [30].

Despite evidence documenting the health risks of stimulant use for MSM, effective treatments for stimulant use disorder are limited—overall and, in particular, the type of MSM-tailored interventions that are likely to be most effective with this population. Existing treatments employ behavioral modification strategies such as cognitive behavioral therapy (CBT) [31–33] and contingency management to reduce stimulant use [34–36]. However, results from studies of MSM entering traditional behavioral treatment for substance abuse suggest that, although reductions in both substance use and HIV-related sexual risk accrue almost immediately upon treatment entry, these effects diminish over time [37, 38]. Moreover, at specialized settings or clinics serving MSM, the dearth of effective treatments as well as difficulties implementing existing evidence-based HIV prevention modalities remain significant barriers to addressing stimulant use and the associated HIV sexual risk behavior of these men.

Behavioral activation (BA) is an evidenced-based approach, developed to treat depressed mood but applied to other related mental health challenges [39–41], that promises to be a useful supplement to HIV risk reduction efforts for MSM with problematic stimulant use. The BA model proposes that life events, which can include trauma, biological predispositions to depression, or the struggles of daily life, over time lead individuals to experience low levels of positive reinforcement [42–45]. Under the BA model, self-defeating behaviors that perpetuate depression, such as stimulant use, may serve the function of coping with negative feelings and make the individual feel better in the short-run; however, these behaviors may ultimately exacerbate depressive symptoms through a process of negative reinforcement. BA, therefore, appears to work to address mood by helping clients learn strategies to re-engage in life by identifying and actively participating in pleasurable, goal-directed activities.

By extending the BA model to the treatment of stimulant use, we hypothesize that, for MSM abusing stimulants, re-engaging in the non-drug using aspects of their life will facilitate their ability to benefit from concomitant HIV risk reduction counseling. The hypothesized mechanism of action is that BA will help MSM re-engage in pleasurable non-drug use activities (e.g., interests or hobbies that were enjoyable before stimulant use) that will serve as a natural reinforcer for functional behavior, improve depressed mood when not using stimulants by experiencing increases in pleasure and mastery, and decrease overall distress so that MSM can reduce their stimulant use and better benefit from HIV risk reduction counseling. Our strategy of developing the intervention is consistent with a staged approach to psychosocial treatment development [46] in that we conceptualized the intervention from formative qualitative work [47] and then openly field-tested this intervention and found it to be feasible to deliver and acceptable to participants and showed significant within person decreases in CAS acts, stimulant use, and depressed mood over 6 months [48]. We subsequently conducted a pilot RCT of this intervention compared to a standard of care (SOC) comparison condition and found that intervention condition participants reported fewer CAS acts with men whose HIV serostatus was either positive or unknown; fewer CAS acts with men whose HIV serostatus was either positive or unknown while under the influence of stimulants; and more continuous days abstaining from stimulant use compared to participants in the control condition.

Drawing on the success of our formative work, aim 1 of the current study is to determine the efficacy of the intervention in a fully powered randomized controlled trial (RCT). Aim 2 is to examine the degree to which reductions in sexual risk are mediated by reductions in stimulant use and increases in pleasurable non-drug use activities as well as other conceptual mediators of the intervention and associated with epidemiologically-identified moderators of sexual risk and stimulant use. Aim 3 is to estimate the cost-effectiveness of the intervention.

## Methods/design

### Overview of design

**Fig. 1:**
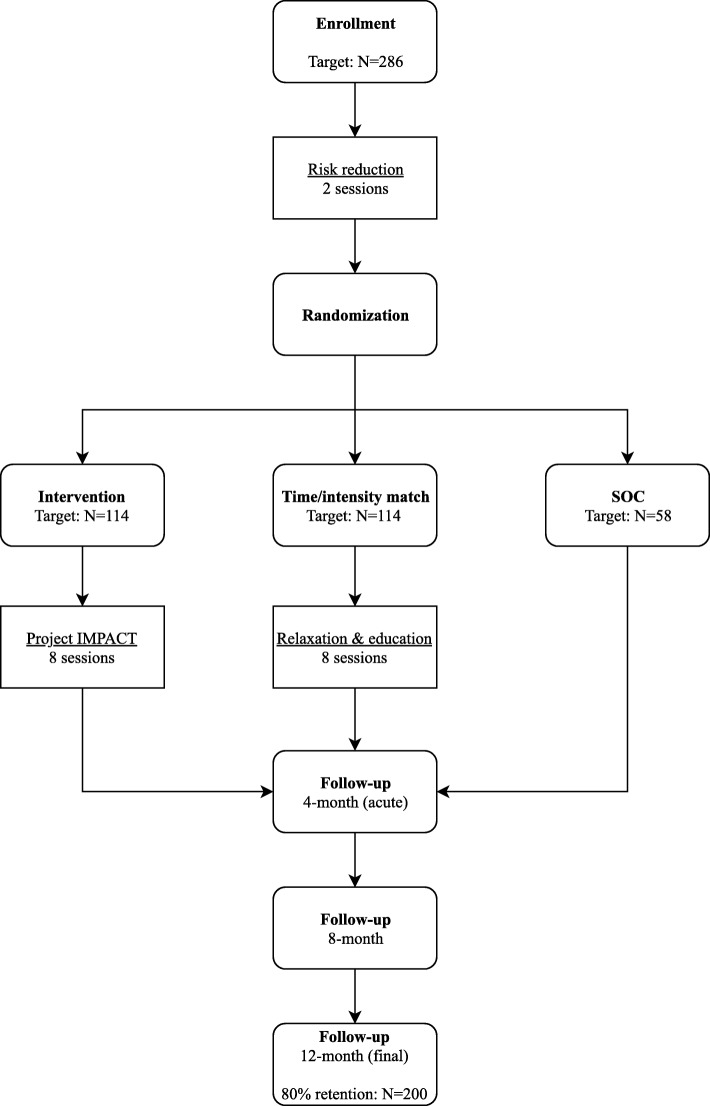
Flow chart of study process

Project IMPACT is a three-arm RCT enrolling 286 HIV-uninfected MSM with stimulant use disorder across two sites in Boston (*N* = 143) and Miami (N = 143). Participants are randomized 2:2:1 into one of three arms: (1) the Project IMPACT intervention, consisting of BA and CBT with HIV sexual risk reduction counseling (*N* = 114); (2) a time- and intensity-matched control including relaxation therapy and educational support with HIV sexual risk reduction counseling (“relaxation condition”; N = 114); and (3) a standard of care (SOC) comparison condition including HIV risk reduction counseling only (*N* = 58). All participants receive repeated standard HIV testing with pre- and post-test counseling and two risk reduction-counseling sessions prior to randomization (described below). Participants who are randomized are followed for 12 months with assessment visits conducted at four-month intervals: 4-, 8-, and 12-months post-randomization (Fig. 1). Participants receive $50 for each assessment visit and $15 for each counseling/intervention session they attend.

### Identification and recruitment of participants

Study participants are recruited using a combination of passive and active approaches. Passive recruitment includes the posting of study flyers and advertisements displayed at local community-based organizations, gay-targeted venues (e.g., cafes, clubs, restaurants), and clinical spaces (e.g., health clinics, HIV testing facilities, substance abuse treatment centers) or printed in gay-friendly magazines and publications. Recruitment is also conducted online by placing ads on websites (e.g., Craigslist), social media sites (e.g., Facebook, Instagram), and mobile apps (e.g., Grindr, Jack’d, Scruff). E-mails are also sent to partner healthcare, advocacy, and community organizations. Participants are also asked to refer friends, co-workers, or acquaintances who may be eligible. Participant inclusion and exclusion criteria are shown in Table 1.

**Table 1:** Inclusion and exclusion criteria

Inclusion Criteria	Exclusion Criteria
• 18 years or older • HIV-uninfected (verified at baseline) • Assigned male sex at birth • Self-identifies as MSM • Self-reports in past 4 months: CAS (receptive or insertive) with male partner while using stimulants (a few hours prior to or during sex), without PrEP^a^ • Able to read, speak, and understand English	• Enrolled in another HIV prevention study (evaluated case-by-case) • Unable to give informed consent due to mental/physical illness, cognitive limitation, or substance intoxication • Unable to give informed consent due active suicidal ideation (may join once resolved) • Lived in Boston/Miami area < 4 months

^a^MSM who are not 100% PrEP adherent are eligible

### Randomization

Following their baseline visit, all participants complete two HIV risk reduction counseling sessions (described below). Upon completion of the second risk reduction session, participants are randomized to 1 of the 3 study arms using block randomization. Specifically, computer-generated randomization assignments are generated at each site, and participants are randomized in blocks according to a 2:2:1 (intervention; time-matched control; SOC) allocation. After randomization, participants randomized to the intervention or relaxation condition return for subsequent sessions the following week; participants randomized to the SOC return for assessment visits only (Table 2).

**Table 2:** Schedule of major assessment points

Assessment	Procedure(s)
Baseline/enrollment	Eligibility screening, informed consent, interview-administered quantitative assessment, self-report battery, rapid HIV testing, toxicology screening (urine), psychiatric diagnostic assessment, blood collection for PrEP testing^a^
4-, 8-, 12-month follow-up visits	Interview-administered quantitative assessment, self-report battery, rapid HIV testing (12-month only), toxicology screening (urine), diagnostic assessment for stimulant use disorder, blood collection for PrEP testing^a^

^a^Only participants who report being < 100% adherent to PrEP

### Assessments visits

Assessments are conducted at baseline and 4-, 8-, and 12-months post-randomization. At baseline, eligible participants complete baseline diagnostic and clinician-rated assessments, administered by study staff, as well as a self-report quantitative assessment battery administered via computer-assisted self-interview (CASI). At post-randomization visits, participants are administered assessments by an independent assessor who is blind to study condition and also complete a self-report battery via CASI (Table 2).

### HIV testing

Participants are tested for HIV at baseline. Those who are randomized are also HIV tested at the 12-month assessment visit. Participants are tested using the FDA-approved OraQuick® ADVANCE™ HIV-1/2 Antibody Test (finger stick; sensitivity: 99.6% [98.5–99.9] and specificity: 100% [99.7–100]; OraSure Technologies, Bethlehem, PA). Since rapid HIV tests are screening tests similar to conventional HIV enzyme immunoassays (EIAs), confirmatory testing is only required for reactive (positive) results. All reactive and indeterminate HIV test results are lab-confirmed via blood draw; those that are confirmed to be HIV-infected at baseline are not randomized.

### Description of the intervention

The Project IMPACT intervention consists of 10 weekly, in-person sessions that include two HIV risk reduction (RR) sessions, two sessions of cognitive behavior therapy for reducing substance use, and six BA sessions (with the final session focusing also on relapse prevention). Each session is delivered by a trained interventionist and lasts approximately 50 min. Throughout all sessions, the interventionist employs a therapeutic stance informed by motivational interviewing (Miller & Rollnick, 1991). The specific modules include:

#### Risk reduction counseling (2 sessions)

Consistent with the IMB model for wellness-promotion [49, 50] the purpose of this module is to (1) promote knowledge about risk reduction related to sexual behavior and substance use; (2) enhance participants’ motivation to engage in less risky behaviors; and (3) support participants as they develop strategies to change their behavior. First, psychoeducation regarding HIV acquisition risk behaviors (information) is provided, including topics such as condomless sex with and without PrEP, and sex in the context of a primary partner with a known undetectable viral load due to antiretroviral therapy adherence. Next, motivation to change sexual behavior is promoted through a non-judgmental exploration of the participant’s sexual history, perceived risks and benefits of current sexual behavior, ideal sexual relationships, limits, and barriers (e.g., motivation or skills) to staying within these limits.

#### Cognitive behavior therapy (CBT) to prepare participants for behavioral activation (2 sessions)

Following the risk reduction sessions, participants in the intervention receive two CBT sessions to help them identify triggers, develop assertiveness and refusal skills, and problem-solve situations that present a high risk for lapses—such as avoiding certain places, managing money, and changing their schedule to build in structure and accountability. In these sessions, the interventionist also provides information about how to differentiate a “lapse” from a “relapse,” and to anticipate difficult or high-risk situations that may trigger drug use and plan for how to successfully navigate them. Participants determine their substance abuse goals and, together, the participant and the interventionist monitor progress towards that goal.

#### Behavioral activation (5 sessions)

In the BA sessions, the interventionist works with the participant to increase the amount of time the participant spends engaged in pleasurable or mastery-inducing activities without using stimulants, which, in turn, work to promote positive mood, reduce the desire to use substances, and bolster the participant’s motivation to engage in sexual risk reduction. Each session begins with a review of instances in which the participant engaged in (or was tempted to engage in) stimulant use or sexual risk behavior, as well as the feelings and behaviors immediately before and after the episode.

Components of problem-solving training [51] are utilized to teach participants how to break down an overwhelming task into manageable steps with the goal of reducing behavioral avoidance and, in particular, breaking the maladaptive and self-perpetuating cycle of stimulant use in response to wanting to improve one’s mood (e.g., participant withdrawal from stimulants, feels depressed/unable to experience pleasure, participant uses more stimulants to feel better, problem worsens, participant engages in continued HIV risk). Consistent with all modules, a motivational [52] style is employed by the interventionist to identify discrepancies between goals and behavior, and help the participant move to a higher level of readiness to change—while maintaining an explicit therapeutic stance that the agency for change resides with the participant.

The interventionist instructs the participant to use a mood and activity-monitoring sheet between sessions to track his daily behavior and note the context and impact of pleasurable, non-drug use activities. During sessions, the pair reviews the activity monitoring sheets, and the interventionist supports the participant in his efforts to incorporate more activities that promote a sense of pleasure and mastery (e.g., volunteering, social events, and other supportive activities), which are core to the efficacy of BA.

#### Review and plan for relapse prevention using behavioral activation (1 session)

The focus of this session is, to the extent possible, transitioning participants to ‘be their own therapist.’ In this final module, the participant and interventionist review skills discussed and lessons learned through prior modules, and engage in problem-solving to address any remaining obstacles blocking the participant’s path to attaining their substance use and sexual risk goals. The interventionist also encourages the participant to take responsibility for his continued progress by using the acquired skills to face future stressors and engaging with ongoing social support or more formal psychotherapy, as needed.

### Time- and intensity-matched control condition “relaxation condition”

Comparing the treatment against a credible time- and intensity-matched control allows us to determine if the intervention is efficacious over and above general counseling with the same intensity and duration (e.g., attention spent with the interventionist vs. the content). Relaxation and educational support are interventions typically employed in psychosocial outcome studies as credible placebo controls [53–56]. The credibility of this condition is supported by the fact that individuals with stimulant use disorder are faced with intense cravings for continued use, and relaxation procedures can assist with decreasing physiological arousal, reducing emotional distress, and helping participants make more effective decisions regarding use. Further, life stressors that elicit strong negative emotions are frequent triggers of substance use; using substances to cope is a common response.

Participants in the relaxation condition also receive the two sexual risk reduction sessions as well as eight weekly therapy sessions focused on relaxation and educational support (for a total of 10 sessions). Each session lasts approximately 50 min. A summary of the two risk reduction sessions is provided above and a summary of the eight relaxation sessions is outlined below:

#### Introduction and preparation for relaxation sessions (1 session)

Following a similar model as the IMPACT intervention, this session is designed to prepare participants to receive the relaxation sessions. During this session, the therapist provides an overview of and rationale for the relaxation and educational support counseling and psychoeducation about stress from stimulant use and stimulant withdrawal.

#### Relaxation and educational support (6 sessions)

Adapted from the Individual Relaxation Treatment Workbook [57] and Cognitive-Behavioral Stress Management for Individuals Living with HIV [58], these sessions involve training participants in progressive muscle relaxation and applying it to various symptoms of stimulant cravings. Relaxation techniques include: diaphragmatic breathing, progressive muscle relaxation, guided imagery, and warming training (i.e., autogenics—desensitization-relaxation technique). These sessions also involve providing support to patients on a weekly basis for whatever difficulties they encounter during the week and provide assistance in using relaxation to cope with these difficulties.

#### Review and plan for relapse prevention using relaxation techniques (1 session)

In this module, the participant and interventionist review skills discussed in prior modules and engage in problem solving to address remaining obstacles blocking the participant’s attainment of their substance use and sexual risk behavior goals. The interventionist also encourages the participant to take responsibility for his continued progress by using the acquired risk reduction and relaxation skills to face future stressors and engaging with ongoing support or psychotherapy, as needed.

### Standard of care comparison condition

The SOC comparison group receives the same two sexual risk reduction counseling sessions as those in the intervention and the relaxation condition, but no additional sessions. After completing the two sessions, these participants return for assessment visits at 4-, 8-, and 12-month follow-ups.

### Measures

Patterns of sexual risk behavior, substance use, and depressive symptoms and cost-effectiveness of substance abuse and mental health treatment are assessed at all visits via a combination of clinician/interview-administered assessments and self-report. Socio-demographics and mental health diagnoses are assessed at baseline only.

#### Primary outcome

Our primary outcome variable is condomless anal sex (CAS) acts without the protection of consistent PrEP use. Specifically, participants are asked to report the number of times (continuous) in the past 4 months in which they engaged in CAS with a male partner whose HIV serostatus was either HIV infected or of unknown status. CAS is assessed via self-report, including CAS in the context of stimulant use. Past four-month PrEP adherence is measured by self-report and verified by testing PrEP levels in participants’ blood. Self-report measures include asking participants to indicate the percent of time that they take PrEP (0–100% in 10% increments) and how many days in the past 4 months that they missed a dose. Participants reporting less than 100% adherence and/or any missed days will be considered not adherent. Adherence verification via blood samples is completed for individuals who report 100% adherence. Blood specimens are collected via venipuncture to create a Dry Blood Spot Card for PrEP quantification. A composite variable of CAS acts that confer risk in the prior 4 months is then created, adjusting CAS acts to “0” for participants with protective levels of PrEP verified by blood test results.

#### Secondary outcome

Stimulant use in the past 30 days is assessed by study staff using TLFB procedures [59]. Using a calendar, participants report retrospective estimates of their daily stimulant use over the 4 months prior to their assessment visit date. Standard TLFB memory aids are used to enhance recall (e.g., key dates serve as anchors for reporting stimulant use).

#### Mediators

Depressive symptoms will be assessed using two measures: the HAM-D [60] and CES-D-10. The HAM-D is one of the most widely used clinical measures of depression and anhedonia in psychiatric research that has strong psychometric reliability and validity [61]. The HAM-D is assessed at baseline by the interventionist and at follow-up by an independent assessor who is blind to randomization assignment. The self-reported 10-item Center for Epidemiologic Studies Depression Scale (CES-D) [62] will complement the assessor-rated HAM-D in that the HAM-D reduces inter-person variability in the rating system, but the CES-D allows for self-report replication. Engagement in positive events (i.e., behavioral activation) will be measured at each assessment visit using the interviewer-administered the Behavioral Activation for Depression Scale (BADS). The BADS [63] is a 25-item scale that specifically assesses the behaviors believed to be responsible for change (i.e., behaviors that should lead to increased contact with response-contingent positive reinforcement) according to the BA treatment model [64]. The scale measures four factors (activation, avoidance/rumination, work/school impairment, and social impairment) with good factor structure, internal consistency, test-retest reliability, and construct and predictive validity.

#### Potential moderators

We will explore potential moderators of intervention effects. These include socio-demographics such as age, race/ethnicity, socio-economic status (e.g., income and education), sexual orientation, relationship status; and psychosocial factors such as HIV risk perception [65], sexual compulsivity [66], concurrent substance abuse treatment (also an indicator of intervention contamination) [67], sexual minority stress [68]; distress tolerance [69]; and social support [70].

#### Cost-effectiveness

The measurement of cost-effectiveness includes an assessment of both study-related costs and participant-related costs. These measures assist in distinguishing if there is differential resource utilization between participant visits by treatment arm. Study-related costs are captured at each visit and follow up, are determined by assessing the amount of time required for the interventionist to deliver the intervention, write chart notes, and obtain clinical supervision. Participant resource utilization is assessed via an interviewer-administered survey at each assessment visit and via self-report at each intervention session for both the intervention group and relaxation condition. Specifically, participants are queried about utilization of inpatient and outpatient services for medical, psychiatric and substance use reasons, and costs associated with these services. Additionally, participants are asked to report the number of stimulant-free days in the past 4 months. The SDS and stimulant-free day questions are interviewer-administered at each major assessment visit.

### Statistical analysis

Initially, the distribution of all variables will be assessed, as will the correlations between all study variables and the primary and secondary outcomes. We will examine patterns of missing data, which are expected to be low, as the study uses computer-assisted interviews, reducing non-response. The primary anticipated reason for missing data is attrition due to loss to follow-up. Based on our prior pilot studies [48, 71], we estimate 20% attrition at 12 months. Outcome variables will be examined to determine which distributional models (e.g., Gaussian, Poisson) are most appropriate for subsequent statistical procedures.

#### Specific aim 1

The primary analysis will compare changes in HIV risk from baseline to the 4-month visit between the intervention and the two control conditions. Similarly, we will compare changes to the 8-month and 12-month visits. We will use generalized linear models (GLM) with properly-chosen link functions (based on the distribution of dependent variable) to analyze longitudinal data for each study aim. The GLMs will be estimated using generalized estimating equations with robust standard error estimates (GEE) [72], which provides an extension of regression analysis to the case of correlated or repeated observations, and allows for the inclusion of both categorical and count dependent variables and for appropriate modeling of covariance structures when observations are correlated across time. With appropriate link functions, GLM can readily handle dependent variables with normal distributions, dichotomous outcomes, count data (Poisson distribution), and overdispersed or zero-inflated count data (negative binomial models). We will follow an intent-to-treat model, analyzing participants in the study arm to which they were assigned regardless of their participation. A sensitivity analysis will compare those who have completed all intervention sessions compared to those who have not. These methods will also be used to determine the longitudinal effects of the intervention on additional outcomes, including decreases in the number of stimulant use episodes, and increases in intervention mediators.

#### Specific aim 2

If the intervention works to reduce sexual risk-taking behaviors among the sample in significantly greater magnitude than the comparison condition, we will assess the extent to which this relationship works through several possible mediators, including reduction in stimulant use, increases in pleasurable (but safe) activities, behavioral risk reduction skills, and reduced depression (which is a stimulant withdraw symptom). For mediation analyses, we will employ MEDIATE procedures [73]. MEDIATE estimates the total, direct, and indirect effects of causal variable(s) (xlist) on the outcome variable (yvar) through a proposed mediator variable or set of mediator variables (mlist), controlling for (optional) one or more variables in (covlist). MEDIATE is similar to INDIRECT [74] but allows multiple X variables, and also offers features for handling and coding a single multicategorical X variable. Inferences for indirect effects can be based on either percentile bootstrap confidence intervals or Monte Carlo confidence intervals. For effect modification (moderation) analyses, we will add interaction terms one-by-one for the intervention condition and the potential moderators (e.g., age, race/ethnicity, psychosocial factors, such as depression). Significant or large interaction terms suggest that the effects of the intervention differ for different subgroups, as defined by the moderators.

#### Specific aim 3

The incremental improvement in quality-adjusted life expectancy associated with the intervention will also be estimated. We will describe the incremental cost-effectiveness ratio (the incremental cost of the intervention compared with the comparison condition divided by the incremental benefit) and the incremental net benefit [75]. The net benefit approach combines both incremental cost and clinical benefit into a single measure and has been recommended when there is a possibility of a negative incremental cost-effectiveness ratio because of the difficulty with interpretation. Because the dollar value of clinical benefit (i.e., days free of stimulant use) is not clearly established, we will use data that has been developed from other studies on patients’ willingness to pay for an additional day free of substance use [76–80]. This threshold will be examined in sensitivity analyses. By incorporating costs and benefits associated with each individual payer, we will have the capacity to demonstrate that the intervention provides good value for money.

### Sample size calculation

The primary power analysis is based on longitudinal, mixed effects modeling comparing HIV risk—CAS acts not protected by PrEP—between the intervention and comparison conditions with repeated measures (of the prior 4 months) at baseline and over 12 months of follow-up. Careful consideration was given to the effect size used in the power analysis and is estimated based on data from our R34 pilot RCT study [71]. In the present study, all three arms will receive SOC HIV pre- and post-test risk reduction counseling at baseline and at their 12-month follow-up assessment visits and, as such, all three arms will likely decrease their HIV risk. Thus, we powered this study using true-scenario effect size data from our pilot RCT showing a mean difference of 4.7 acts of CAS between the intervention vs. control condition [2.24 (3.94) vs. 6.94 (9.89) *p* < .00001]. Although our pilot study had a large effect on reductions in sexual risk behaviors, effect sizes from small studies like our pilot study are less reliable and frequently inflated [81]. Additionally, in the present study, the time- and intensity-matched and SOC comparison groups will be receiving two risk reduction sessions and, as such, they will likely also decrease their engagement in CAS. Therefore, we powered the study to detect a 20% (or greater) difference in the rate of change in sexual risk between groups over the course of the study, expecting all three groups to show improvements from baseline. A 20% or greater difference in number of CAS acts has been previously described as a clinically meaningful change [82] related to HIV sexual risk outcomes. The proposed longitudinal analyses, which will simultaneously analyze CAS acts not protected by PrEP at each of the follow-up visits, increases the power to detect an effect. Based on these data, with a power of 85% and alpha level of 0.05, approximately 230 completers of the proposed interventions would allow for testing the difference between BA-RR intervention (*N* = 114) vs. (1) Relaxation time-match control (N = 114) and (2) SOC control (*N* = 58) and plan to enroll 286 (to account for a potential 20% attrition at the month 12 visit). We anticipate that adjustment of CAS acts for rates by PrEP protection will be equal across arms (~ 10%) and is built into our estimates above.

## Discussion

We describe herein the design of the Project IMPACT, an RCT testing the efficacy of a novel, individually-delivered, behavioral intervention to reduce sexual risk behavior and stimulant use among HIV-uninfected MSM at high risk of infection. The intervention employs evidence-based BA and CBT strategies combined uniquely with HIV risk reduction counseling to reduce sexual risk behavior by increasing pleasurable but safe life activities without drug use. The study has several strengths, including a focus on problematic stimulant use in MSM; the use of an evidence-based behavioral treatment to boost the effects of traditional therapies (e.g., CBT and risk reduction counseling); and the utility of its therapeutic approach and rigorous research design that utilizes two comparison conditions.

The IMPACT intervention is particularly innovative as it is designed to reduce MSM’s HIV risk behavior through reductions in stimulant use and improved mood. MSM have some of the highest rates of HIV infection in the U.S. [1, 2], which is driven in part by problematic substance use among this population [7–12]. Stimulant use is endemic to urban MSM in the U.S. [3–6] and has been shown to increase MSM’s risk for engaging in sexual risk behavior [22–28]. Despite the documented association between stimulant use and HIV risk behavior among MSM [29, 30], few interventions target sexual risk taking in MSM with problematic stimulant use [31–36], and none have demonstrated long-term efficacy [37, 38]. One potential contributor to the intractability of stimulant use is that continued stimulant use may be exacerbated, in part, as a result of feelings of anhedonia between substance use episodes, and the fact that existing treatments lack adequate attention to replacement activities or the role of depressed mood as a relapse trigger [42–45], which is the cornerstone of the BA approach [39–41].

Therefore, we designed and tested a treatment approach that combines BA with other common, evidence-based treatment approaches to address the loss of pleasure resulting from chronic stimulant use by helping MSM to re-engage with pleasurable activities. We hypothesize that, by targeting the loss of pleasure—and providing standard risk reduction counseling and enhancing skill building to reduce stimulant use and engage in safer sex behavior—the intervention will be effective at reducing HIV risk behavior and concurrent stimulant use and equip MSM to maintain these reductions and prevent relapse over time.

Although there is some promise with mirtazapine—a noradrenergic and specific serotonergic antidepressant—for stimulant use reduction [83], currently, there are no FDA-approved medications for the treatment of stimulant use disorder [84]; thus, new behavioral treatments represent an important strategy to intervene on stimulant use and HIV sexual risk among MSM. One major criticism of many behavioral interventions designed and tested under ideal research conditions is the limited utility and feasibility of implementing these treatments in real-word settings [37, 38]. Recognizing this limitation, we designed a brief, 10-session intervention that can be implemented by substance abuse counselors without significant specialized training, thus increasing the likelihood that the intervention can be easily disseminated to community settings if found to be effective. Additionally, the content, structure, and format of the intervention were informed, developed, and refined through forums that involved stimulant-using MSM as well as input from community advisory boards, thereby ensuring that MSM stimulant users lived contextual realities are addressed in a manner to promote sexual safety, and further increasing the utility of the intervention in community care settings.

Finally, while the intervention was designed with the feasibility of employment in mind, we still ensured that a methodologically rigorous approach to testing the efficacy of the intervention was used. Specifically, our use of a three-arm design allows us to determine if the treatment is efficacious above general risk reduction counseling or therapeutic approaches delivered with the same intensity and duration (e.g., attention spent with the interventionist vs. the content). Our design also allows for the assessment of incremental cost-effectiveness. Once the cost of the intervention is known relative to other treatment therapies, this data can be used by policymakers and healthcare administrators to determine whether this treatment should be funded and employed in community settings over other treatments. Together, our research design and therapeutic approach highlight the rigor of this study and the potential utility of the intervention for real-world use.

The intervention described here could fill an important treatment gap. If successful, the intervention will be tested in translation studies to examine its effectiveness and feasibility of dissemination into real-world healthcare, mental health, and social service settings with the ultimate goal of providing community clinicians with an effective tool to reduce stimulant use and curb HIV spread.

## Abbreviations

ACASI: Audio computer-assisted self-interview
BA: Behavioral activation
BA-RR: Behavioral activation with hiv risk reduction
CAS: Condomless anal sex
CBT: Cognitive behavioral therapy
DSM-5: Diagnostic and statistical manual of mental disorders, 5th edition
HIV: Human immunodeficiency virus
MSM: Men who have sex with men
PrEP: Pre-exposure prophylaxis
RCT: Randomized controlled trial
SOC: Standard of care
TLFB: Timeline follow-back
